# Audiovisual Content to Promote Women Scientists on the YouTube Channels of Spanish Biosanitary Research Institutes

**DOI:** 10.3390/ijerph18189698

**Published:** 2021-09-15

**Authors:** Javier Almela-Baeza, Beatriz Febrero, Antonio Pérez-Manzano, Adrián Bonache-Ibáñez, Pablo Ramírez

**Affiliations:** 1 Faculty of Communication and Documentation, University of Murcia, 30100 Murcia, Spain; javier.almela@um.es; 2 Department of Surgery, Pediatrics, Gynecology and Obstetrics, Instituto Murciano de Investigaciones Biosanitaria IMIB-Arrixaca, School of Medicine, University of Murcia, 30100 Murcia, Spain; ramirezp@um.es; 3 Department of Evolutionary Psychology and Education, University of Murcia, 30100 Murcia, Spain; aperez@um.es (A.P.-M.); adrian.bonache@um.es (A.B.-I.)

**Keywords:** YouTube, visibility of women scientists, audiovisual postproduction, science and media, visual communication

## Abstract

YouTube is an appropriate social network for disseminating scientific audiovisual content, and this content can help to make the position of women in science, and gender equality, more visible. The aim of this study is to analyse the visibility of women scientists on the YouTube channels of Spanish biosanitary research institutes accredited by the Instituto de Salud Carlos III. A mixed study of the channels and communication departments of the institutions has been carried out, analysing metrics of audience impact, the type of audiovisual production and the use of YouTube in the institution. Of the 1914 videos analysed, 4% (*n* = 76) of the content is related to the visibility of women scientists and has little impact. The audiovisual production is basic and informative, without a dramatic narrative structure and focused on transmitting the personal experiences of women scientists. It is necessary to invest resources in institutions to improve the production and dissemination of content that makes women in the scientific field more visible, especially among students.

## 1. Introduction

The YouTube social platform, or network, was founded in February 2005 by three US engineers with the initial idea of sharing videos of a social nature among users. Currently, YouTube belongs to the company Google, and is the content-sharing platform with the greatest impact on society. Given the way it is used, it is meant to democratize the production of audiovisual content among citizens, overthrowing the giants in the world of communication and production of audiovisual content [1]. This platform has not only produced a user who consumes, shares and comments on content, known as prosumers, but it has also opened the door to companies and institutions that see in YouTube an appropriate means of sharing content related to their activity [2].

The quality of the audiovisual production of science-related content on YouTube has led to conflicting opinions among researchers and users. Some believe that this content created by non-professional users could lack interest and rigour [3]. In the field of scientific reporting, on the other hand, there are many researchers who assign positive value to this means of communication, given that it encourages a collaborative culture [4].

A range of studies have analysed the impact of science-based audiovisual content on society [5,6,7]. The YouTube platform has more than 4000 channels related to science and scientific reporting, and on these channels, there are more than 100,000 items about this matter, mainly created by YouTubers, universities, governmental institutions and prosumers [8]. 

Many institutions use YouTube to create an audience that wishes to be regularly informed about their activity [9]. This way of sharing information is also well-accepted by users, especially when it is about prosocial, scientific and technological subjects [10,11,12,13]. In the ambit of scientific reporting, studies have been carried out analysing the perception of users who consume YouTube (who are more receptive to the content created by prosumers) of the content produced by institutions, and above all those related to science content [14]. A study undertaken in England on young people aged between 13 and 18 years revealed that teenagers follow YouTubers because of the ease with which they can find health-related information topics compared to using institutional channels [15]. This issue raises the need for online content to be overseen by communication and healthcare professionals to correctly train the users who consume this content [16]. 

A recent study carried out by Muñoz Morcillo et al. [17] has analysed the narrative, aesthetic and audiovisual post-production characteristics of the content of scientific reporting on an international level. This study highlights the fact that scientific audiovisual content usually consists of short videos of wide-ranging genres, foremost among which are documentaries, animated films and monologues. The audiovisual production of this content is not complex; it is carried out by professionals and uses classic recording techniques. As for editing and infographics, a high level of complexity has been found, with a notably good quality of postproduction sound. Another one of the characteristics highlighted by this study is that the digital content is characterised by having introductory and ending sequences, or high-quality headings and advanced postproduction. As far as the main conclusion is concerned, they indicate that the main feature of the production of this content is centred on the closeness of the characters, who are experts in telling stories and in making a connection with the spectator [17].

According to McLuhan, the potential of this means of communication on the configuration of society is a crucial factor in training and information [18]. McLuhan understands that the way in which we receive information has a greater influence on the user than the information itself, which can be extrapolated to a present situation where communication channels are becoming more extensive while also affecting the way in which we access information and the context of the message [19]. The limited representation of women in traditional fields of communication has been analysed in several studies [20,21,22]. This tendency continues to exist when we talk about their presence or interaction in social networks, where gender stereotypes are still being found [23]. The relationship between scientific reporting and women is not found among the paradigms of users of the YouTube social network, where other topics are very popular such as humour, sports or gameplay, in which men tend to be the protagonists [24,25]. Gender differences in STEM degrees is a reality [26]. A recent study has suggested that women still face social prejudice and discrimination in Science, Technology, Engineering and Mathematics (STEM), above all when it comes to reporting audiovisual content in social networks. This has a crucial effect on the viewers and on the popularity of women scientists online [27]. In spite of this historical marginalisation, several studies are analysing the presence of women in the media and this situation is being reversed [28].

In Spain, the Instituto de Salud Carlos III (ISCIII) is responsible for accrediting national biosanitary research institutes [29]. These centres of international reference bring together research and researchers who are the most relevant in Spain. What is more, they are responsible for passing on their findings to society through communication channels. At present, there are 32 accredited biosanitary research institutes, of which 21 have a YouTube channel for broadcasting audiovisual content.

The objective of the present study was to analyse audiovisual content related to the visibility of the role of women scientists on the YouTube channels of biosanitary research institutes in Spain. The specific objectives included: to analyse the impact of content on the audience, to discover the type of audiovisual production and to evaluate the use of YouTube in communication departments within the institutes.

## 2. Materials and Methods

### 2.1. Sample Selection

A selection was made of the audiovisual content that dealt with subject matter aimed at developing the visibility of women scientists in research or whose main and explicit objective was to encourage the role of women in scientific matters. Data collection of the content of the different channels was carried out during the month of January 2021 in all the biosanitary research institutes accredited by the ISCIII which had a YouTube channel, including the following institutes: the Marqués De Valdecilla Research Institute (IDIVAL), the Hospital Universitari Vall D’hebron Research Institute (IR-HUVH), the Gregorio Marañón Healthcare Research Institute (IiSGM), the Biodonostia Healthcare Research Institute (IIS BIODONOSTIA), the Sant Pau Biomedical Research Institute (IIB SANT PAU), the Illes Balears Healthcare Research Institute (IdiSBa), the August Pi Y Sunyer Biomedical Research Institute (IDIBAPS), the Sevilla Biomedicine Institute (IBIS), the Germans Trias I Pujol Health Sciences Research Institute (IGTP), the Lérida Biomedical Research Institute (IRB LÉRIDA), the Salamanca Healthcare Research Institute (IBSAL), the La Fe University Hospital Research Foundation (IIS LA FE), the Valencia Hospital Clinic Healthcare Research Foundation (INCLIVA), the Maimónides Biomedical Research Institute of Córdoba (IMIBIC), the La Paz Hospital Healthcare Research Institute (IDIPAZ), the Virgen de la Arrixaca Biosanitary Research Institute of Murcia (IMIB), the Aragón Healthcare Research Institute (IIS Aragón), the Málaga Healthcare Research Institute (IBIMA), the Bellvitge Biomedical Research Institute (IDIBELL), the Granada Biosanitary Research Institute (ibs. GRANADA) and the Biocruces Bizkaia Healthcare Research Institute (IIS BIOCRUCES).

### 2.2. Experimental Design

The study was divided into two parts:

#### 2.2.1. An Analysis of the Impact of Audiovisual Content about the Visibility of Women Scientists, the Type of Audiovisual Production Used and Its Relationship with the Rest of the Content on the YouTube Channel

In order to determine which items were related with the visibility of women scientists, two members of the research group who are experts in Audiovisual Communication carried out an exploratory qualitative analysis of the content produced by the institutions. We selected the content in which the main theme was the visibility of women scientists.

In order to measure the impact of this content, the metrics of user interaction provided freely by the YouTube platform were used, and using these data as a starting point, a descriptive quantitative study of the audiovisual production was carried out. In each channel, we analysed the overall interaction metrics and the metrics for each item of content individually. With regard to the metrics of interaction with the user and the channel in general, we identified the following variables:

- Number of subscribers
- Number of videos
- Total number of viewings of the content in each YouTube channel

Regarding the variables related to the metrics of interaction with the user of each specific genre of content, we should highlight the following notable findings:

- Viewings of each type of content
- Average duration
- Number of “likes”
- Number of “dislikes”
- Comments made by the users

A classification was made of the selected content paying attention to the audiovisual production. In order to do this, we took into account the classification created by Muñoz Morcillo et al. in their study entitled “Typologies of the popular science web video” [17]. Considering the following variables:

- Production
- Type of lighting
- Editing technique
- Sound
- Special effects (VFX)
- Cinematography
- Types of shot
- Narrative strategies
- Cinematographic genre
- Topic
- Number of actors according to gender

#### 2.2.2. An Analysis of the Use of YouTube by Communication Departments in ISCIII-Accredited Institutes

The second part of the study analysed the importance of YouTube for heads of communication in the institutes and how they deal with the production and reporting of content when it is about the visibility of women scientists. In order to determine these data a questionnaire was administered with the following questions:

- The importance of YouTube in their communication strategy
- Frequency of the publication of content on YouTube
- Topics covered in the content and the frequency with which it is shared
- The target audience at which the content is aimed and the level of responsibility of the institution for reporting content aimed at students
- Level of responsibility of the institution for reporting content encouraging the equality of women in scientific fields

### 2.3. Measurement Instrument

In order to find out the opinion of the heads of communication in the institutions that participated in the study, a form was administered through the Google Forms platform. The study was conducted according to the guidelines of the Declaration of Helsinki and approved by the Ethics Committee of the University of Murcia, protocol code 3359/2021 and date of approval 20 May 2021.

The data related to the metrics of interaction with the users of content related to the institutions were obtained from the information freely offered by YouTube and accessible to any user. 

## 3. Results

Of the 1914 items of content registered, 4% (*n* = 76) were items aimed at encouraging equality in the field of science or the promotion of the figure of scientific women among students.

### 3.1. Interaction of the User with the Content of the YouTube Channels

In Table 1 the following information is shown: the number of subscribers, the number of videos and the total number of viewings of the channel in each institution. 

Sixty-two percent of the channels (*n* = 13) had one or more items of content aimed at promoting the visibility of women scientists. Twenty-three percent (*n* = 3) of the channels with content about the visibility of women scientists had more than 20% of the total items referring to the visibility of women scientists. The visibility of women scientists on one of the channels was greater than the general average of viewings of the rest of the content, as observed in Table 1.

With regard to the content about the visibility of women scientists, most was created as a result of International Women’s Day and Girls in Science. The average duration of the content about the visibility of women scientists was 7 min 30 s. When considering the means with which the user could assess the content it was observed that there was a mean of 13 likes per item, and only 5 dislikes were registered in total. There were no comments from registered users on this type of content, as we can appreciate in Table 2.

### 3.2. Typology of the Audiovisual Production of Content about the Visibility of Women Scientists

#### 3.2.1. Production, Illumination, Editing, Sound and VFX

Ninety-five percent (*n* = 72) of the items of audiovisual content analysed had a professional audiovisual production quality. Regarding editing, 88% of the items were created using a professional editing process, compared to 12% (*n* = 9) which were uploaded directly to the channel after being filmed on a camera. The number of shots in audiovisual content can determine the author’s messaging intent. When a video had more than three shots, we interpreted that the author intended to construct an actual film; this can also occur with the editing of one or two shots, unless they are a documentary or of an informative nature. Sixty percent (*n* = 46) of the videos had three or fewer shots and were of an informative nature. All the items were put together without using advanced editing techniques such as time lapse, slow motion, jump cut, etc. The video format was of a good quality, 87% (*n* = 66) were produced in Full HD 1080P. Regarding sound, 87% (*n* = 66) were of a good quality, both narrations and statements, 68% (*n* = 52) did not use music and the rest used ambient music in accordance with the subject matter.

Eighty-seven percent (*n* = 66) were recorded in an indoor location without a setting, and the remainder were recorded indoors with a setting. With regard to lighting, all used ambient lighting and no interior lighting systems were seen.

Twenty-five percent of the items of content (*n* = 19) did not have an introduction, seven percent (*n* = 5) started with a headline, 36% (*n* = 27) started with a static corporate logo and 5% (*n* = 4) began with an animated logo. The most widely used VFX techniques were text in picture, used in 87% (*n* = 66), mainly to identify the character, and 36% (*n* = 27) used basic 2D graphics techniques.

#### 3.2.2. Cinematography and Types of Shots

The shooting techniques were quite advanced and appropriate for any kind of production. Ninety-five percent of the videos (*n* = 74) were recorded using a tripod; of the others, two were recorded using a webcam and two using a static mobile phone.

The most common shot was the medium close up (MCU), used in 88% of the content (*n* = 67), with the classic shot being used in documentaries or news stories with testimonials. The next most common type of shot used was the long shot (LS), which was used most often to contextualise the scene; of the videos, 26% (*n* = 20) used this type of shot. Another identified shot was the wide-angle shot, used to show groups of people; it was identified in 12% of the content analysed (*n* = 9). Finally, the detailed shot, used to describe elements, was used in 14% of the content (*n* = 11).

#### 3.2.3. Narrative, Generic and Topic-Based Strategies

Ninety-seven percent of the content was narrated in the first person so that the interlocutor receives the information from the hand of the protagonist, establishing a direct connection with the spectator. Complex narrative structures have not been identified; there were no subplots.

With regard to the topics, 86% (*n* = 65) were personal non-fictional testimonials or experiences. Twenty-eight percent (*n* = 21) appealed to collaboration from the spectator and the need to visualise the role of women in science, and 13% (*n* = 10) based their opinion on focusing on future women scientists with the slogan “you can do it”.

The most common genre was the “interview”, used by 67% (*n* = 52), followed by the “publicity spot” used by 11% (*n* = 8). The “monologue” genre was used on two occasions and three of the items were “reports”.

#### 3.2.4. Number of Actors According to Gender

In the following table (Table 3) we can see the number of actresses and actors, according to age groups, distributed among the 76 videos.

### 3.3. The Importance of the YouTube Platform for the Institutions

Of the 21 accredited biosanitary institutions with a YouTube channel, 86% (*n* = 18) completed the questionnaire.

**Figure 1:**
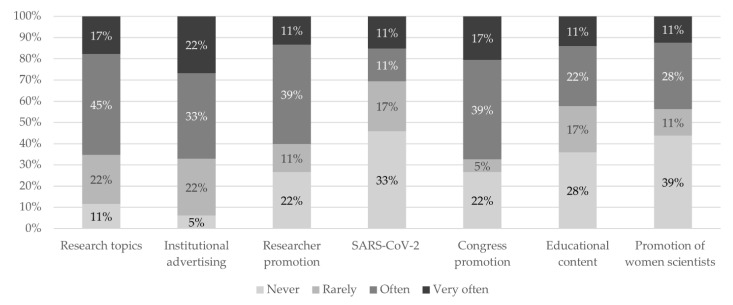
Topics of the most common content and the frequency with which it is uploaded to the channel. Source: our own creation.

Seventy-eight percent (*n* = 14) of the heads of communication from the institutions surveyed indicated that YouTube was normal or not very relevant in their communication plan. With regard to the use they make of the platform, 61% (*n* = 11) indicated that they uploaded audiovisual content every month, compared to 28% (*n* = 5) who stated that they posted content every week; the remaining 11% (*n* = 2) stated they had the platform but did not use it. In the following figure (Figure 1) the contents have been grouped according to the topic and frequency with which the department of each institution reports sharing content related to that topic.

With regard to the age of the population targeted with content by the institutions, 89% of the departments (*n* = 16) stated that their content is aimed at people in the age range of between 18 and 29 years and those from 30 to 45 years, 83% stated that they aim their content at the public aged between 45 and 65 years of age and 33% (*n* = 6) indicated that their content could be aimed at those aged under 17 years.

When we consider whether the institution should encourage the creation of content aimed at education, 62% (*n* = 11) stated that this was necessary, but they did not have enough resources to produce and promote it.

Regarding whether there is an institutional responsibility to broadcast content to help increase women scientists’ visibility in order to achieve parity with male scientists and provide equality of opportunities, 39% (*n* = 7) indicated that they do this but that their production and broadcasting should be enhanced, 33% (*n* = 6) stated that it was important but that they did not have enough resources to produce it and 28% (*n* = 5) stated that it was important and they produced it. Of the institutions analysed, only one had a specific section and playlist aimed at the creation of audiovisual content for promoting females in science.

## 4. Discussion

The YouTube social network or platform is a communication channel with a considerable level of impact, appropriate for scientific reporting in general and for reporting content related to the visibility of women scientists and the visibility of women in scientific fields [5,6,7]. The content of the institutions analysed was of a high quality and was very rigorous in its messaging, as found in the studies analysed [1,5,6,7]. It is created by professionals in the field of audiovisual production, and the scientists themselves are the protagonists of this content, offering credibility to the viewer given that the origin and solvency of the information obtained can be identified [16].

The content analysed according to genre is focused on passing on the experience of scientists, the need to the make women scientists visible and encouraging young women to choose science subjects in their studies, thus encouraging a reduction in the gender gap with regard to the choice of STEM careers [30]. The departments of communication do not produce much content aimed at promoting the role of women in science, and the content that is produced does not have much of an impact if it is compared with the rest of the content from the different institutional channels and the content produced by YouTuber scientists in Spain. The YouTuber scientists have much higher viewerships, or numbers of subscribers, as in the example of José Luis Crespo’s «QuantumFracture» channel, which has the greatest reported impact on matters related to science in Spain with a total of 2,730,100 subscribers and 201,081,158 viewings when the data from this study were gathered [31]. These are very high figures if we compare them with the 2990 subscribers and 1,599,118 viewings found on the channel with the greatest impact in this study. The main reason why the heads of communication of this study stated that they had a small amount of content with limited impact is the lack of funding and infrastructure to produce and share this type of content.

Morcillo et al. analysed 190 videos of considerable impact related to scientific reporting [17]. The study indicated that the content with the most impact was classified into different types of film genres, but the content analysed in the present study is mainly of an informative/documentary nature focused on testimonials, particularly those related to the visibility of women scientists. As indicated in the study by Morcillo et al., the content is of a good quality in terms of audiovisual production, while the content analysed is also of a good standard, using classic production and editing techniques and VFX, and very rarely using either specially created scenes or professional lighting for the production. In terms of the production, these videos are basic, and a more advanced production would help to achieve content with a greater impact. In the narrative, we do not find complex structures and the videos are not developed from a dramatic standpoint; the content is more informative/testimonial. Therefore, although the impact on the viewer is positive, the content does not have the potential to have a significant impact or provide a reason to share the content on a mass scale. A reasonable alternative could be the creation of videos featuring a YouTuber scientist, which would help the content reach a younger population that prefers to consume this type of content from their peers [15]. 

For the heads of the departments in the institutions analysed, YouTube is not a very important network in the communication plan. The publication of content is generally discreet, once a month, as is the number of subscribers to their channels. Regarding the subjects the content of their channels are divided into, the promotion of women and science would be in fifth place, which leads us to believe that the interest in making the role of women in science more visible is rather limited, although the heads of department state that it would be necessary to invest more in this area. The most important target population in terms of the visibility of women in science is the group aged under 17 years, in order to encourage young women to undertake university and vocational studies in STEM subjects. Nevertheless, the results of this study suggest that this is not the target population in the current institutional communication plans, in spite of the recommendations to disseminate these kinds of messages in order to enhance gender equality in STEM careers [30]

The institutional use of YouTube is low if we compare it with other studies that have analysed the impact of similar content in the field of science [31]. The main reason given for this finding is the lack of funding and infrastructure for achieving a higher number of subscribers and a greater amount of content.

### Limitations of This Study

Although the institutions analysed are bringing together lines of research and the most relevant researchers in Spain, there are other institutions that could be analysed, thus widening the sample and diversifying the results. In addition, the qualitative analysis of the heads of communication in the institutions analysed could have been more thorough by carrying out a structured interview or focus group.

## 5. Conclusions

For the institutions of biosanitary research in Spain accredited by the ISCIII, the reporting of audiovisual content related to the visibility of women in the field of science is important, but the content produced is limited and does not have much impact on society. 

The production of this content is professional but carried out using basic narrative production methods of an informative nature, with limited dramatic input. An improvement in these aspects would allow the content to have a greater impact, so long as it is correctly broadcast on social media.

The production of educational content focused on a target population of school age would increase the number of subscribers and viewers of these channels, and would also encourage the reporting of content aimed at making women more visible in the world of science.

## Figures and Tables

**Table 1:** Metrics of interaction with the user of the channel in general and with the content about the visibility of women scientists.

Institution	Total Videos	Videos about Visibility of Women Scientists	Total Viewings	Average Viewings	Average Viewings–Visibility of Women Scientists	Number of Subscribers
IR-HUVH	705	11 (1.5%)	636,969	905	307	Not defined
IDIVAL	499	0	1,599,118	3205	0	2990
IDIBELL	140	8 (5.7%)	46,114	329	106	296
IMIB	110	3 (2.7%)	19,524	177	767	175
IMIBIC	83	1 (1.2%)	21,264	256	256	334
IDIBAPS	72	27 (37.5%)	20,871	290	119	112
INCLIVA	67	16 (23.8%)	15,239	227	179	138
IIS LA FE	46	3 (6.5%)	6129	133	124	Not defined
IRB LÉRIDA	40	1 (2.5%)	3131	125	21	165
IBSAL	23	0	14,418	627	0	Not defined
IGTP	21	1 (4.7%)	2175	104	100	15
IBS. GRANADA	19	0	24,391	1284	0	43
IBIS	19	0	4478	257	0	55
IDISBA	17	1 (5.8%)	1382	81	14	30
IBIMA	15	1 (6.6%)	6012	401	67	10
IIS BIODONOSTIA	13	1 (7.7%)	5868	451	13	22
IDIPAZ	11	0	304	28	0	15
IIS BIOCRUCES	8	2 (25%)	733	92	39	7
IIB SANT PAU	3	0	2350	783	0	0
IIS ARAGON	2	0	769	385	0	13
IISGM	1	0	262	262	0	4

Source: our own creation.

**Table 2:** Interaction of the user with the content about the visibility of women scientists.

Institution	Videos about the Visibility of Women Scientists	Likes	Dislikes	Comments	Mean Duration (min:s)
IR-HUVH	11	33	0	0	1:01
IDIBELL	8	21	0	0	4:29
IMIB	3	19	0	0	8:21
IMIBIC	1	1	0	0	10:02
IDIBAPS	27	27	2	0	1:50
INCLIVA	16	48	0	0	10:30
IIS LA FE	3	24	3	0	37:36
IRB LÉRIDA	1	1	0	0	0:29
IGTP	1	1	0	0	1:05
IDISBA	1	0	0	0	0:52
IBIMA	1	2	0	0	5:41
IIS BIODONOSTIA	1	0	0	0	11:56
IIS BIOCRUCES	2	0	0	0	4:56

Source: our own creation.

**Table 3:** Number of actresses and actors and videos in which they appear grouped by ages.

Age Range	Actresses	Actors
>17	4 actresses in 2 videos	0
18–35	90 actresses in 29 videos	3 actors in 1 video
36–55	132 actresses in 68 videos	12 actors in 4 videos
55–70	24 actresses in 15 videos	2 actors in 2 videos
>70	0	0

## Data Availability

Data are contained within the article.
